# Redox Balance Keepers and Possible Cell Functions Managed by Redox Homeostasis in *Trypanosoma cruzi*

**DOI:** 10.3389/fcimb.2019.00435

**Published:** 2019-12-20

**Authors:** Andrea C. Mesías, Nisha J. Garg, M. Paola Zago

**Affiliations:** ^1^Instituto de Patología Experimental, Consejo Nacional de Investigaciones Científicas y Técnicas (CONICET) - Universidad Nacional de Salta, Salta, Argentina; ^2^Department of Microbiology and Immunology, Institute for Human Infections and Immunity, University of Texas Medical Branch, Galveston, TX, United States

**Keywords:** *Trypanosoma cruzi*, stage-specific oxidants, antioxidant network, regulation, redox-dependent mechanisms

## Abstract

The toxicity of oxygen and nitrogen reactive species appears to be merely the tip of the iceberg in the world of redox homeostasis. Now, oxidative stress can be seen as a two-sided process; at high concentrations, it causes damage to biomolecules, and thus, trypanosomes have evolved a strong antioxidant defense system to cope with these stressors. At low concentrations, oxidants are essential for cell signaling, and in fact, the oxidants/antioxidants balance may be able to trigger different cell fates. In this comprehensive review, we discuss the current knowledge of the oxidant environment experienced by *T. cruzi* along the different phases of its life cycle, and the molecular tools exploited by this pathogen to deal with oxidative stress, for better or worse. Further, we discuss the possible redox-regulated processes that could be governed by this oxidative context. Most of the current research has addressed the importance of the trypanosomes' antioxidant network based on its detox activity of harmful species; however, new efforts are necessary to highlight other functions of this network and the mechanisms underlying the fine regulation of the defense machinery, as this represents a master key to hinder crucial pathogen functions. Understanding the relevance of this balance keeper program in parasite biology will give us new perspectives to delineate improved treatment strategies.

## Introduction

Chagas disease (ChD) is one of the most important neglected tropical diseases in South and Central America and in Mexico. It is estimated that 6–7 million people are infected with *Trypanosoma cruzi* (*T. cruzi*), and ~300,000 new cases of ChD emerge each year that account for >10,000 deaths *per* year^1^ During last two decades, ChD cases have also been reported in non-endemic countries (e.g., United States and Canada), Western Pacific region, and Europe (reviewed in Schmunis, 2007; Albajar-Vinas and Jannin, 2011; Tanowitz et al., 2011), primarily due to immigration of seropositive individuals from endemic countries. However, in the Southern US, the natural cycle of *T. cruzi* transmission is evidenced with the detection of high rate of infection in dogs (Curtis-Robles et al., 2017, 2018) and autochthonous cases of ChD in humans (Garcia et al., 2017).

Two drugs, benznidazole and nifurtimox, are currently available for the treatment of patients diagnosed early after *T. cruzi* infection, but these drugs have limited efficacy in the chronic disease phase (Morillo et al., 2015). Further, these drugs have several side effects, and are not recommended for persons with neurological and psychiatric disorders or some degree of kidney failure, and for pregnant women (reviewed in Patterson and Wyllie, 2014). Several vaccines are in the experimental stage (reviewed in Rodríguez-Morales et al., 2015; Rios et al., 2019) even though none of these are yet available to prevent or control human *T. cruzi* infection. Thus, new prophylactic and therapeutic strategies for control of *T. cruzi* infection and chronic ChD are urgently needed.

*T. cruzi* is an intracellular kinetoplastid parasite with a complex life cycle that goes through several biochemical and morphological changes during its transit through the vector and mammalian host. Remarkably, *T. cruzi* can potentially infect 1000's of vertebrate species, and at least 40 invertebrate species (Teixeira et al., 2009). This parasite's tremendous adaptability to infect a wide variety of hosts ensures its survival in the sylvatic and domestic cycles. An example of the pathogen's plasticity can be found in the reactive species management system used by *T. cruzi* to keep homeostasis and ensure redox-dependent pathways.

## *T. cruzi* Exposure to Oxidants in Insect Vectors

### Oxidant Stressors in the Triatomine Vectors

**Figure 1:**
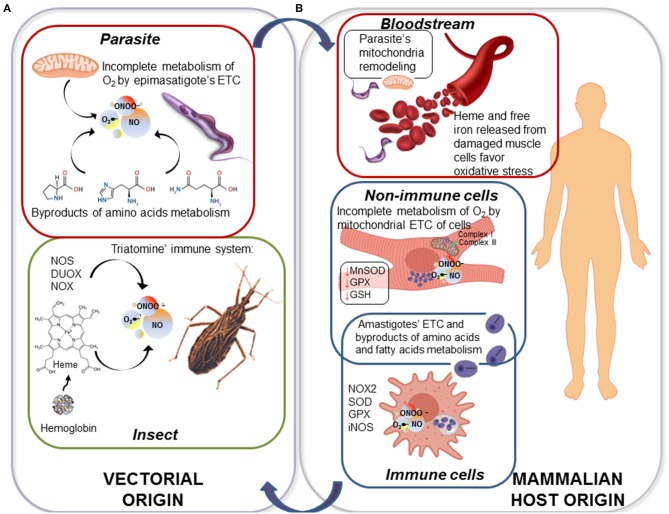
ROS and RNS are produced throughout the life cycle of *Trypanosoma cruzi*. **(A)** Triatomines respond to *T. cruzi* infection and replication by up regulation of nitric oxide synthase (NOS), dual oxidase (DUOX), and NADPH oxidase (NOX) that directly produce nitric oxide (NO) and superoxide radical (O_2_•^−^); and NO and O_2_•^−^ may produce highly stable hydrogen peroxide (H_2_O_2_) and highly toxic peroxynitrite (ONOO^−^). Further, upon uptake of blood meal by the insect, epimastigotes are exposed to great amounts of heme, a highly oxidant molecule. Parasite itself generates endogenous oxidative molecules produced by its electron transport chain (ETC) and the oxidation of amino acids as carbon source. **(B)** When metacyclic trypomastigotes are transferred from bug to mammalian host, they first encounter the ubiquitous oxidants present in bloodstream, e.g., hemoglobin and Fe^2+^ released from damage cells. When parasite invades the immune cells, such as macrophages, it is exposed to a stronger oxidative/nitrosative stress sustained by inducible NOS (iNOS) and NOX2 in phagolysosome. As an intracellular amastigote, parasite replicates in the host cell cytoplasm and generates ROS, primarily because it prefers oxidative metabolism of amino acids and fatty acids over glycolysis. If parasite gets to infect a non-immune cell (e.g., cardiac myocyte), ROS continue to be present due to mitochondrial dysfunction of ETC at complex I and complex III.

*T. cruzi* faces a variety of oxidative stressors of internal and external origin during its replication and differentiation in the insect (Figure 1). Briefly, after being ingested by triatomines with a blood meal, parasite goes through an active binary division as an epimastigote in the insect gut. Once the nutrients available for parasite proliferation become limited, epimastigote forms move to the posterior midgut, adhere to the wax cover of the rectal cuticle by hydrophobic interactions, and undergo metacyclogenesis. During parasite's replication and passage through the vector's intestinal tube, significant amount of oxidants are produced by the triatomine's immune system (Figure 1A) (Ursic-Bedoya and Lowenberger, 2007). It is suggested that triatomine recognition of pathogen-associated molecular patterns (PAMPs) triggers innate immunity as well as humoral and cellular protection (reviewed in Azambuja et al., 2016). Briefly, the gut lumen is a prime site for the production of immune effectors including reactive oxygen species (ROS) and reactive nitrogen species (RNS) (Garcia et al., 2007, 2010; Genta et al., 2010) by a pool of enzymes. Nitric oxide synthase (NOS) was firstly identified through its cross-reaction with human NADPH oxidase p67^phox^ antibody (Whitten et al., 2001), and its expression and activity in *R. prolixus* was later confirmed by 2,3-diaminonapthalene fluorescence-based assay that detects NO (Whitten et al., 2007). Other enzymes, namely dual oxidase (DUOX) and NADPH oxidase (NOX), produce superoxide radical (O_2_•^−^), and the latter can dismutate to hydrogen peroxide (H_2_O_2_) or react with nitric oxide (NO) to produce peroxynitrite (ONOO^−^) (Ha et al., 2005; Azambuja et al., 2016). The phenoloxidase (PO) cascade, a hallmark immune component of insects, is also present in triatomines leading to the production of toxic quinones, melanin, and some intermediates of reactive oxygen species (ROS) and reactive nitrogen species (RNS) to encapsulate and kill the pathogens (reviewed in Flores-Villegas et al., 2015). PO activity was increased after vector infection with *T. cruzi* Dm28c strain though it had no effect on parasite viability (Castro et al., 2012), and therefore, its function as a driver of innate immunity against *T. cruzi* was not established. Moreover, these authors postulated that parasite-induced PO modifies vector immune responses to decrease the gut microbiota and favor parasite development in the insect gut.

In addition to vectorial oxidants, epimastigotes and metacyclic trypomastigotes are exposed to other compounds like heme (ferriprotoporphyrin-IX) and heme breakdown products in the midgut of the insect; produced by degradation of hemoglobin that is the most abundant protein in mammalian blood. At an estimated concentration of 10 mM blood heme (bound to hemoglobin), triatomine midgut is believed to carry toxic amounts of heme during digestion of a single blood meal (Graça-Souza et al., 2006). Free heme, even at 50–100 μM concentration, is a toxic molecule due to its ability to generate ROS (Gutteridge and Smith, 1988) catalyzing the oxidation of proteins, the formation of cytotoxic lipid peroxides via lipid peroxidation and damaging DNA (reviewed in Kumar and Bandyopadhyay, 2005). Nevertheless, triatomine vectors exploit heme crystallization into hemozoin as a prime redox regulator mechanism that protects the insect itself, but also promotes parasite infection (Ferreira et al., 2018). Therefore, in this context, free heme toxicity would be mitigated. *T. cruzi* genome lacks the genes/proteins required for the biosynthesis of heme (El-Sayed et al., 2005); however, it is an essential cofactor that *T. cruzi* must intake from the host. It was demonstrated that heme and its breakdown products promote proliferation in *T. cruzi* epimastigotes (Lara et al., 2007). Strikingly, *T. cruzi* insect stage is able to withstand 1 mM hemin—a concentration known to disrupt phospholipid membranes—without any obvious toxic effect (Lara et al., 2007). Nogueira et al., also described that heme proliferative phenomenon was accompanied by a marked increase in ROS formation in epimastigotes (Nogueira et al., 2011, 2015). It was proposed that heme-induce transient oxidative stress drives the epimastigote proliferation through activation of calcium-calmodulin-dependent kinase II (CaMKII) (Souza et al., 2009; Nogueira et al., 2011). Thus, *T. cruzi* epimastigote can avoid heme toxicity, and instead use it as a signaling molecule for its proliferation in the insect midgut (Lara et al., 2007; Paes et al., 2011; Nogueira et al., 2017).

### Oxidants Produced by Insect Stage Parasite

*T. cruzi* also produces oxidants during its replication and differentiation in the insect midgut. ROS production in *T. cruzi*, in general, is closely related to nutrients metabolism in each developmental stage. The epimastigote stage relies on oxidation of L-proline, L-histidine, and L-glutamine for energy supply. These amino acids are released from digestion of blood proteins being abundantly present in the hemolymph and tissue fluids of the hematophagous vectors (Cazzulo, 1984; Bringaud et al., 2006). L-alanine, is produced as a metabolic end product by *T. cruzi* when it grows in a medium rich in glucose and amino acids, but it can also be taken up and oxidized to CO_2_ delivering electrons to electron transport chain (ETC) (Girard et al., 2018). Thus, amino acids oxidation fuels respiratory complexes and oxidative phosphorylation for ATP generation; however, during this process, electron leakage to O_2_ can also result in O_2_•^−^ formation, possibly supporting an oxidative environment (Figure 1A). Further, Nogueira et al. (2017) showed that presence of heme during *in vitro* culture of *T. cruzi*, induced mitochondrial membrane hyperpolarization and an increase in endogenous O_2_•^−^ production. As we mentioned previously, the heme-induced mitochondrial ROS were beneficial in promoting epimastigote survival and proliferation. Conversely, urate-like antioxidants produced in the hemolymph were found to arrest epimastigotes' growth and promote differentiation of epimastigotes to metacyclic infective form (Nogueira et al., 2015). To sum up, *T. cruzi* epimastigotes appear to utilize the components of oxidative stress for their growth in the insect gut, and then for switching to metacyclogenesis and be ready for transition to mammalian infective stage.

## *T. cruzi* Exposure to Oxidants in the Mammalian Host

### Exposure to Oxidative and Nitrosative Stress in Immune Cells

Triatomines release infective, non-replicative, metacyclic trypomastigotes in feces while taking the next blood meal on a mammalian host. Once trypomastigotes enter the blood stream of the vertebrate host, they quickly infect a variety of cells and differentiate to the replicative amastigote stage. During this process, the parasite has to deal with a second wave of oxidative stress. Studies in mice and humans show that innate and adaptive immune responses, involving macrophages, neutrophils, natural killer cells, B and T lymphocytes, should control the parasite through the production of ROS/RNS, proinflammatory T_H_1 cytokines, trypanolytic antibodies, and cytotoxic T lymphocytes' activity; readers are referred to excellent reviews on this topic (Junqueira et al., 2010; Machado et al., 2012; Cardillo et al., 2015; Bonney et al., 2019). A thorough analysis of all the components of natural and experimental innate and adaptive immunity against *T. cruzi* infection is beyond the scope of this article. Herein, we will focus on macrophages that offer the first line of defense upon parasite engulfment (discussed in Lopez et al., 2018).

*T. cruzi* (metacyclics and trypomastigotes) can actively invade a variety of non-immune and immune cells and also may be phagocytosed by macrophages and dendritic cells (reviewed in Walker et al., 2013). The parasite uptake triggers an almost immediate increase in the expression of inflammatory cytokines followed by delayed and subpar production of O_2_•^−^ and NO in macrophages (Koo et al., 2018). As in the vector host, in infected macrophages also, inducible NADPH oxidase (NOX2) produces O_2_•^−^ that can be transformed spontaneously or enzymatically by superoxide dismutases (SODs) to H_2_O_2_, and the latter is dismutated by GPx and catalase (Figure 1B) (Gupta et al., 2011). Likewise, inducible nitric oxide synthetase (iNOS) produces NO that can react with O_2_•^−^ and generate ONOO^−^ in infected macrophages. Although ONOO^−^ has a short life, it is the most powerful cytotoxic effector produced by macrophages for parasite killing (Alvarez et al., 2011). Yet, it must be mentioned that the proinflammatory cytokines and the oxidative and nitrosative stress are capable of controlling, but not preventing the dissemination of virulent parasite strains from macrophages (reviewed in Lopez et al., 2018; Koo and Garg, 2019). This is, in part, attributed to the ability of the virulent isolates of *T. cruzi* to orchestrate their antioxidant system and allow sub-par and delayed activation of oxidative/nitrosative burst in the host immune cells (Piacenza et al., 2009b; Zago et al., 2016). Others have indicated that oxidative stress produced in response to *T. cruzi* infection correlates with higher parasite burden in an animal infection model, pointing to oxidative environment as an enhancer of infection (Paiva et al., 2012). In agreement with this finding, treatment with compounds, such as iron chelator desferrioxamine and melatonin that also have antioxidant capacity reduced the parasite proliferation (Arantes et al., 2007; Santello et al., 2007). However, it was a correlative observation, and authors did not clarify if the observed proliferative effects on the parasite were indeed due to antioxidant nature of these drugs. Regardless, the current literature indicates a dual role of ROS/RNS in parasite control vs. parasite proliferation and spreading in the mammalian host. Immune oxidative response can, in fact, control parasite infection whereas at lower levels, an oxidant environment may promote pathogen replication.

### Exposure to Oxidative Stress in Non-immune Cells

Parasite is also exposed to cytotoxic molecules in non-immune cells, primarily because a wide variety of ROS and RNS are continuously formed as byproducts of aerobic metabolism. In general terms, mitochondrial ETC coupled with oxidative phosphorylation accounts for 85–90% of the O_2_ consumed in a cell. Up to 3% of the consumed O_2_ is incompletely metabolized, and results in O_2_•^−^ release (Silva et al., 2011; Wang and Hai, 2016). The heart is particularly dependent on mitochondria to produce the energy required for its contractile activity, and mitochondria represent up to 30% of the total volume of cardiomyocytes, providing 90% of the cellular ATP energy through oxidative phosphorylation. In cardiomyocytes infected by *T. cruzi*, and in the myocardium of chronically infected animals (Mukherjee et al., 2003; Wen and Garg, 2004) and ChD clinically symptomatic patients (Cunha-Neto et al., 2005; Wen et al., 2006; Wan et al., 2012; Dhiman et al., 2013), mitochondrial dysfunction was well-documented by us and other researchers. Specifically, activities of the respiratory complex I and complex III were compromised and resulted in a significant increase in electron leakage to O_2_ and O_2_•^−^ production in mitochondria of infected cardiomyocytes and ChD hearts (Vyatkina et al., 2004; Gupta et al., 2009). *In vivo* studies in mice and rats showed that mitochondrial defects persisted beyond the acute phase of infection, correlating with high mitochondrial ROS (mtROS) levels during chronic disease phase (Wen et al., 2008, 2017; Wan et al., 2016). Further, the increase in mtROS production correlated with a decline in the expression and activity of the mitochondrial antioxidant enzyme Mn^+2^ superoxide dismutase (MnSOD), and a decline in the cytosolic glutathione peroxidase (GPx) activity and GSH content in the myocardium of chronically infected animals (Wen and Garg, 2004) and in ChD patients (Pérez-Fuentes et al., 2003; Wen et al., 2006; de Oliveira et al., 2007; Wan et al., 2012; Dhiman et al., 2013), thus revealing the persistence of a pro-oxidant milieu along the infection process. These studies point to the role of ETC as an important source of oxidant species in non-immune cells, especially in cardiomyocytes that are one of the main target cells invaded by *T. cruzi* (Figure 1B). Garg and co-workers have proposed that a lack of appropriate antioxidant and repair response result in self-perpetuating mitochondrial dysfunction and ROS production in the heart (reviewed in Lopez et al., 2018; Bonney et al., 2019). This mtROS production in Chagas heart can provide a defense against parasite persistence; however, it also signals the fibrotic gene expression and contribute to evolution of chronic cardiomyopathy (Wan et al., 2012; Wen et al., 2017).

### Non-enzymatic Oxidative Stress

As in the insect stage, *T. cruzi* is also exposed to the non-enzymatic oxidative species in the mammalian host. For example, essential metals, such as iron, zinc, and copper play a critical role in many biological processes. The Fe^+2^/Fe^+3^ and Cu^+2^/Cu^+3^ act as electron donor/acceptor and play a vital role in catalysis of a variety of enzymatic reactions that involve an electron transfer. Specifically, up to 70% of the body iron is present in red blood cells (RBCs) in the form of hemoglobin and in muscle cells as a component of myoglobin (Winter et al., 2014). In these tissues, iron is also present in iron-sulfur (Fe-S) clusters, and it serves as a cofactor to support ETC, respiration, and oxygen transport (reviewed in Rouault, 2012). Likewise, copper is the cofactor of metabolic enzymes (e.g., cytochrome c oxidase in mitochondria, CuZnSOD in cytosol) and it catalyzes the enzymatic activity of key enzymes of the secretory pathway (reviewed in Polishchuk and Lutsenko, 2013; Baker et al., 2017). However, these metals are redox active, and in an oxidative environment undergo redox-cycling reactions leading to exacerbated production of free radical species. The toxicity of iron, and, by association, of copper, is driven by their ability to reduce peroxides, via Fenton chemistry, into highly reactive hydroxyl radical that subsequently reacts at diffusion-limited rates with various biomolecules (Valko et al., 2016; Sánchez et al., 2017). Further, Fe-S clusters are preferred targets of O_2_•^−^, and their oxidation leads to Fe^+2^ release that then can feed Fenton reaction and ROS production (Fridovich, 1995).

Heme and free iron released by dying RBCs and damaged muscle cells may also generate oxidative stress (Beard, 2001; McCord, 2004). Thus, it could be speculated that the breakdown of metal ion homeostasis can expose *T. cruzi* to iron (and up to some extent copper) in the blood stream as well as in muscle cells and tissues that are the preferred site of *T. cruzi* replication. Skeletal muscle and heart may also accumulate considerable amounts of iron as is noted in brain, liver and other tissues, and there is evidence that increased iron storage correlates with ROS formation in tissues (Yoshiji et al., 1992; Barollo et al., 2004; Shoham and Youdim, 2004; Sullivan, 2004). Cytosolic ferritin (binds up to 4,500 iron atoms for storage) may be a source of free iron when it undergoes degradation in response to stress (reviewed in Philpott et al., 2017). Thus, we propose that endogenous iron storage in muscle cells and tissues may also expose intracellular amastigotes to oxidative stress. Still, many reports describe the requirement for a mild oxidizing environment for the efficient iron mobilization, which in turn enhances intracellular parasite growth. Hence, it is suggested that depletion of intracellular iron stores in host cells could impair *T. cruzi* replication. Conversely, host responses transferring iron to the intracellular sites of *T. cruzi* replication may enhance parasite pathogenicity (reviewed in Andrews, 2012; Paiva et al., 2018). Future studies are needed to clearly define the role of iron metabolism and redox signaling in parasite proliferation.

### ROS as a Byproduct of Trypomastigote/Amastigote Metabolism

ROS generation within *T. cruzi* is closely related to available energy sources. In contrast to epimastigote forms of *T. cruzi* that rely on amino acids metabolism in the vector host, infective trypomastigotes have access to abundant glucose (up to 5 mM) in bloodstream of the vertebrate host. Not surprisingly then, trypomastigotes utilize glucose as a preferred carbon source for their energy requirement in the mammalian host (Silber et al., 2009). Glucose fermentation in trypomastigotes' glycosome—a peroxisome-like organelle—produces CO_2_, succinate, and acetate (reviewed in Bringaud et al., 2006; Michels et al., 2006), and provides substrates for oxidative phosphorylation. The bioenergetics metabolism in the intracellular, replicative, amastigote stage remains controversial. Some investigators have indicated that the intracellular form of the parasite arrests the expression of glucose transporters (Silber et al., 2009), and utilizes fatty acids and amino acids taken from the cell cytosol to satisfy its energy needs (Engel et al., 1987; Silber et al., 2009). Indeed, expression of enzymes needed for fatty acids oxidation are increased in amastigote form of the parasite (Atwood et al., 2005), thus suggesting a metabolic shift. However, recent studies have employed a metabolic labeling approach to show that *T. cruzi* infection modulates host cell metabolism and stimulates cellular glucose uptake that can then be utilized by parasite for its own replication in the host cytosol (Shah-Simpson et al., 2017). These seemingly contradictory results could simply suggest that intracellular parasites fuel their metabolism in a flexible manner. Thus, while epimastigotes rely on amino acids catabolism and mitochondrial respiration, trypomastigote—and likely amastigote—parasite forms depend mostly on glycolysis but sparingly use fatty acids or other sources of energy. If such is the case, then they do not need to face a strong endogenous ROS stress that otherwise is associated with high mitochondrial activity.

Nevertheless, from a different line of evidence, Gonçalves et al. (2011) have shown, through functional assessment of mitochondrial metabolism, an increased generation of H_2_O_2_ in the bloodstream trypomastigotes compared to the epimastigote stage (Gonçalves et al., 2011). According to these authors, during the parasite transformation from the insect to infective trypomastigote form, mitochondrial organelle undergoes remodeling that results in (a) increased activities of the respiratory complex II and complex III facilitating electrons' entrance to the ETC, and (b) decreased expression and activity of complex IV. This would promote the generation of an “electron bottleneck,” favoring O_2_•^−^ and H_2_O_2_ formation (Gonçalves et al., 2011). In this scenario, it is argued that even if trypomastigotes are more dependent on glycolysis than oxidative phosphorylation for their energy need (Bringaud et al., 2006; Silber et al., 2009), they may still be exposed to excessive oxidative stress due to mitochondrial remodeling and increased ROS production (Gonçalves et al., 2011).

### Drugs-Derived Oxidative Stress

Treatment with anti-parasite drugs, benznidazole (BZ) and nifurtimox (NFX), also exposes the parasite to oxidative stress. BZ is the preferred drug for the treatment of acute ChD. In this sense, it is worthy of note that several BZ delivery platforms and formulations are been assayed in pre-clinical studies (Scalise et al., 2016; Santos Souza et al., 2017; García et al., 2018) and a pediatric formulation of BZ is available since 2011^2^, improving dosing accuracy, safety, and adherence to treatment. Both BZ and NFX are pro-drugs that are cleaved to their active form in the parasite by nitroreductase type I (NTR-I) (Hall et al., 2011; Hall and Wilkinson, 2012). It is suggested that the activated BZ and NFX (and their metabolites), through direct binding to trypanothione (T[SH]_2_, N^1^,N^8^-bisglutathionylspermidine) and GSH, make these antioxidants unavailable for the parasite's defense (Maya et al., 1997; Trochine et al., 2014). Thus, BZ/NFX, at appropriate concentration, would decrease the availability of low MW thiols such that oxygen redox cycling-derived free radicals, drug-derived free radicals, and reduced electrophilic metabolites are accumulated and cause cytotoxic effects on the parasite biomolecules (Maya et al., 1997). The activation of BZ by NTR-I also generates DNA-toxic glyoxal adducts in an oxygen-insensitive reaction (Hall and Wilkinson, 2012). In the host, BZ promotes an adaptive response to oxidative injury activating the nuclear factor-erythroid 2-related factor-2 (Nrf2) and multidrug resistance associated protein 2 (MRP2) (Rigalli et al., 2016). However, anti-parasite therapy is not always successful because of varying degrees of susceptibility of different parasite strains. This is probably, due to diverse mechanisms such as differences in the thiol content, NTR-I downregulation (Wilkinson et al., 2008), or deletion of copies of the gene encoding prostaglandin F2α synthase (referred as old yellow enzyme) that is involved in activating the anti-parasite drugs (Murta et al., 2006). Nevertheless, at present, there are no drugs clinically superior to NFX or BZ.

## The Complexity of ROS and RNS Cytotoxic Effects on *T. cruzi*

Before we discuss the cytotoxic effects of oxidative stress on parasite, it should be mentioned that ROS and RNS are broad terms that include free radical and non-radical active species with different levels of toxicity. Effect of each active molecule depends on its amount, reactivity, half-life, diffusion properties, and biological interactions. Furthermore, it is well-established that many of these species can give rise to secondary oxidants with different reactivity and lifespan than the initial ones (reviewed in Halliwell and Gutteridge, 2015). Overall, high concentrations of reactive species produced by immune response, oxidative metabolism, and other environmental factors create a complex landscape of oxidative stress that the parasite has to deal with to maintain redox homeostasis for its survival in diverse hosts.

One of the main targets of ROS is DNA. There are many types of oxidative modifications noted in the sugar-phosphate backbone and nitrogenous bases of DNA, and considering the low reduction coefficient of guanine, this base is recognized as the most susceptible nucleotide (David et al., 2007). Once guanine is oxidized, it assumes a *syn* conformation called 8-oxoguanine (8-oxoG) that mimics thymine, and thereby exerts a strong mutagenic effect by transversion. Oxidized DNA is also susceptible to nicks, and introduces single and double strand DNA breaks (Cheng et al., 1992; Aguiar et al., 2013). While double strand breaks are lethal if left unrepaired, oxidized bases may be mutagenic, cytotoxic or both. Indeed, recent studies have shown that exposure to H_2_O_2_ caused a 2-fold increase in the frequency of mutations to *T. cruzi* DNA (Torres-Silva et al., 2018), and it turned out to be cytotoxic to the pathogen (Zago et al., 2016; Mesías et al., 2018). However, others have proposed that oxidative DNA damage, along with the recently documented 8-oxoG lesion repair system in *T. cruzi* (Machado-silva et al., 2016), were beneficial in enhancing the genetic diversity and favored the selection of parasites capable of evading immune system and develop drug resistance (Torres-Silva et al., 2018). Further studies will be necessary to elucidate the levels and types of oxidative DNA modifications that are cytotoxic to *T. cruzi* vs. those that offer survival advantages to the parasite.

Reactive lipid species (RLS) within the membranes can be formed enzymatically (e.g., by lipoxygenase and cyclooxygenase) or through non-enzymatic lipid peroxidation and nitration pathways (Gaschler and Stockwell, 2017). Major classes of RLS include lipid aldehydes, α,β-unsaturated carbonyls, and nitroalkenes (discussed in Esterbauer et al., 1991; Ayala et al., 2014; Gaschler and Stockwell, 2017). Some of these are stable, and can diffuse through cell membrane affecting membrane functions, such as lipid–lipid interactions, ion gradients, fluidity and permeability, thus, causing a major issue for cell homeostasis (Skouta et al., 2014; Van der Paal et al., 2016; Agmon and Stockwell, 2017; Agmon et al., 2018). Other RLS, e.g., 5-deoxy-delta-12,14-prostaglandin J2 (15d-PGJ2), signal intracellular receptor PPAR-γ to mediate resolution of inflammation (Agmon and Stockwell, 2017). There is scarce evidence regarding the generation of RLS and their role in *T. cruzi*; though some investigators have reported that heme-induced ROS elicited lipid peroxidation, 4-hydroxy-2-nonenal (4-HNE) adducts, and yet it supported epimastigotes' proliferation (Nogueira et al., 2011).

Proteins are also major targets of oxidation due to their high concentration and reactivity with multiple oxidants (reviewed in Davies, 2016). Proteins may undergo oxidative modifications that are reversible (e.g., sulfenylation, nitrosylation, or s-glutathionylation of cysteine residue) or irreversible (e.g., protein carbonylation and 3-nitrotyrosine formation); and these oxidative modifications can affect protein function or protein-protein interaction (Cai and Yan, 2013). Alternatively, self-protein oxidative modifications could be exploited by the parasite for signaling various pathways. Indeed, it was shown that in presence of extracellular matrix that constitute the cell surrounding layer crossed by the parasite for invasion of host cells, pathogen undergoes selective S-nitrosylation and tyrosine nitration (Pereira et al., 2015). These authors proposed that *T. cruzi* actively modulates its nitrosylation allowing its internalization into host cells.

In summary, the complete extent of the effects of ROS/RNS on *T. cruzi* is not fully disclosed. Yet, the killing of *T. cruzi* by oxidative stress in immune cells and the generation of DNA-toxic glyoxal and oxidative adducts by BZ are well-documented (Maya et al., 2003; Wilkinson et al., 2011). Thus, the antioxidant enzymes expressed by *T. cruzi* are essential for providing defense against oxidative damage and allow the parasite to thrive in oxidative conditions, discussed below.

## A Distinctive Antioxidant System in Parasitic Protozoans

### Trypanothione (T[SH]_2_), a Unique Derivative of Glutathione

*T. cruzi* antioxidant machinery relies on a low molecular weight diglutathionyl-spermidine conjugate named T[SH]_2_ that is analogous to glutathione (GSH) in most eukaryotes (Manta et al., 2013) (Table 1). The production and consumption of T[SH]_2_ antioxidant is increased in bloodstream and intracellular stages of the parasite (Ariyanayagam and Fairlamb, 2001; Ariyanayagam et al., 2003). Firstly, biosynthesis of GSH substrate in protozoan parasites is catalyzed by two enzymes. Gamma-glutamylcysteine synthetase (GshA) ligates L-glutamate and L-cysteine to form γ-glutamylcysteine; and in a second step glutathione synthetase (GshB) forms a C-N bond between γ-glutamylcysteine and L-glycine to produce GSH. The fluctuating concentrations of GSH in human patient's plasma and the significant decrease in plasma GSH levels in seropositive patients (Dhiman et al., 2013) suggest that *T. cruzi* synthesizes its own GSH as well as utilizes host's GSH to supply the needed substrate for T[SH]_2_ production. Next, the trypanothione synthetase (TryS) enzyme catalyzes an ATP-dependent two-step reaction in which two GSH molecules and a polyamine (spermidine) are used to synthesize T[SH]_2_ in cytosol of the parasite (Oza et al., 2002). The T[SH]_2_ molecules deliver reducing equivalents to peroxidases and other enzymes responsible for neutralizing ROS, and the oxidized form of trypanothione is reduced by trypanothione reductase (TryR) in presence of NADPH produced by the pentose phosphate pathway (PPP) (discussed below and reviewed in Irigoín et al., 2008). Considering their essential function in maintaining reduced T[SH]_2_, TryS, and TryR are attractive drug targets for control of trypanosomes.

**Table 1:** Trypanothione and glutathione are the major, low MW thiols utilized by *T. cruzi* to keep redox homeostasis.

	**Glutathione**	**Trypanothione**
A. Chemical structure Oxidized state:	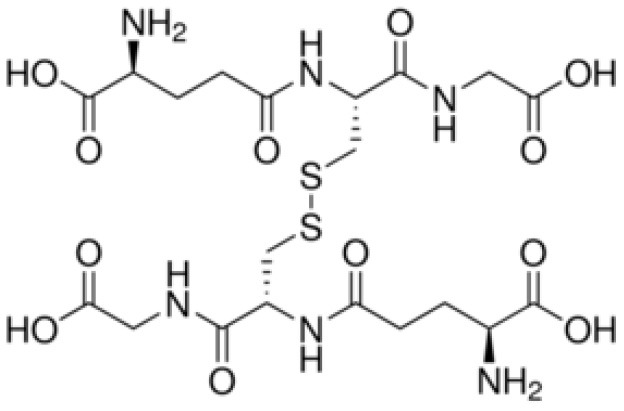	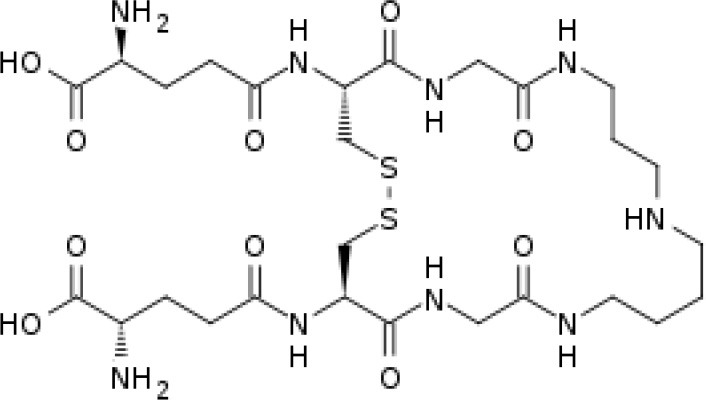 Número de envío [A
Reduced state:	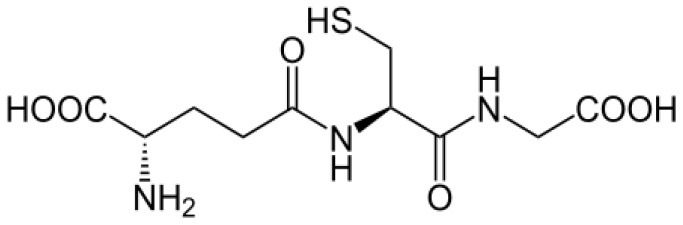	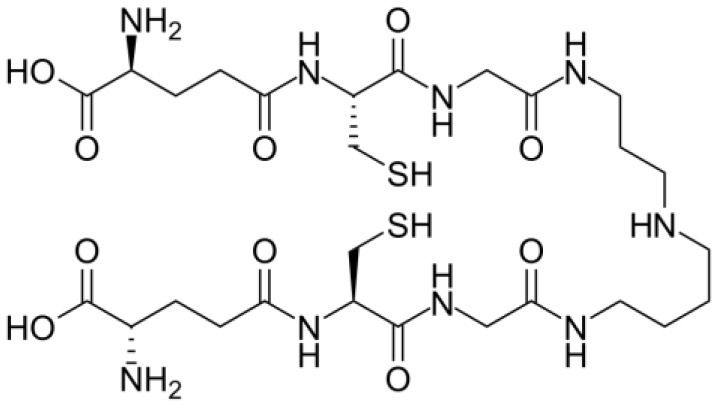 3552373]
B. Enzymes involved	GshA and GshB	TryS
C. Substrates	ATP L-Glutamate L-Cysteine L-Glycine	ATP Spermidine GSH (*possibly synthesized by parasite)
D. Reduction coefficient (Fairlamb and Cerami, 1992)	Eo = −0.230	Eo = −0.242 V
E. pKa (Moutiez et al., 1994)	8.7	7.4
F. Concentration	1 mM in blood (Richie et al., 1996). 6.9 mM in intracellular milieu of HeLa cells (Montero et al., 2013)	0.12–0.64 nmol/10^8^ epimastigotes; 0.25–0.95 nmol/10^8^ bloodstream trypomastigotes; 0.12 nmol/10^8^ amastigotes (Ariyanayagam and Fairlamb, 2001; Ariyanayagam et al., 2003)

*Glutathione (ubiquitous monothiol among eukaryotes) and trypanothione (a kinetoplastid-specific dithiol) are synthesized by parasites' antioxidant machinery, and these molecules play a key role in distribution of reducing equivalents along the antioxidant network. Even when they have similar characteristics, T[SH]_2_ is considered a kinetically superior antioxidant*.

The redox potentials of T[SH]_2_ (2,242 mV) and GSH (2,230 mV) are very similar, though T[SH]_2_ (pKa 7.4) and GSH (pKa 8.7) exhibit dramatic differences in their pKa values (Moutiez et al., 1994). Since rate constants for thiol-disulfide exchange is optimal when the thiol pKa value is equal to the solution pH value, T[SH]_2_ is more reactive than GSH under physiological conditions. Furthermore, dithiols are kinetically superior than the monothiols (Dormeyer et al., 2001) in reducing intramolecular disulfides (Table 1). Thus, it is believed that T[SH]_2_ serves as a major antioxidant molecule in trypanosomes and GSH is primarily consumed to produce T[SH]_2_ in *T. cruzi* (Olin-Sandoval et al., 2012).

Besides T[SH]_2_ and GSH, insect stage epimastigotes utilize a significant amount of *N*1-methyl-4-mercaptohistidine or ovothiol A as antioxidant. Ovothiol A has non-enzymatic reactivity with H_2_O_2_; yet its neutralizing activity is considerably less than that of T[SH]_2_. Therefore, under physiological conditions, it is unlikely that ovothiol A plays a major role in the metabolism of hydrogen peroxide (Ariyanayagam and Fairlamb, 2001). *T. cruzi* also possesses significant levels of vitamin C (ascorbate) antioxidant. Unlike humans and other vertebrates, parasite genome encodes for complete ascorbate biosynthesis pathway, and it is speculated to be localized in glycosome (Wilkinson et al., 2005) and serve as an alternative ROS scavenger in *T. cruzi* and other trypanosomes (Wilkinson et al., 2002a).

### A Network of Antioxidant Enzymes in *T. cruzi*

Trypanosomes utilize a highly developed network of peroxidases and superoxide dismutases to control ROS and RNS (reviewed in Irigoín et al., 2008; Piacenza et al., 2009a; Machado-silva et al., 2016). Briefly, trypanosomes have equipped their mitochondria, the site of electron release to oxygen and O_2_•^−^ formation, with a solid antioxidant defense. These include (a) two iron-dependent, superoxide dismutases (FeSODA and FeSODC) that convert O_2_•^−^ into H_2_O_2_; and (b) a two-cysteine mitochondrial tryparedoxin peroxidase (mTXNPx) capable of rapidly detoxifying H_2_O_2_ and ONOO^−^ (Ismail et al., 1997; Piñeyro et al., 2011a; Phan et al., 2015). The endoplasmic reticulum (ER) utilizes oxidizing environment for the formation of the disulfide bonds required for the folding of proteins into functionally active forms (Malhotra and Kaufman, 2007). Persistent oxidative stress can result in protein unfolding and cell death (Sano and Reed, 2013). Whether trypanosomes face excessive oxidative stress in the ER is not experimentally documented, yet two distinct peroxidases are localized in the ER in *T. cruzi*, namely, ascorbate peroxidase (APx) that utilizes ascorbate to remove H_2_O_2_, and non-selenium glutathione peroxidase II (nsGPx-II) that detoxifies hydroperoxides (Wilkinson et al., 2002c). Others have shown that APx is localized on the ER and mitochondrial membranes in all parasites stages but also on plasma membrane in infective stages (Hugo et al., 2017), thus implying that APx may play a role during parasite invasion. Similarly, trypanosomes utilize a highly specialized peroxisome, labeled as glycosome, for important metabolic functions, including glycolytic pathway, pentose-phosphate pathway, β-oxidation of fatty acids, purine salvage, and biosynthetic pathways for pyrimidines, ether-lipids and squalenes (Haanstra et al., 2016). Accordingly, *T. cruzi* has evolved to localize FeSODB2 and nsGPx-I antioxidant enzymes in the glycosomal compartment (Wilkinson et al., 2000, 2002b; Patel et al., 2010). It was demonstrated that nsGPxs do not metabolize H_2_O_2_, yet, nsGPx-I and -II overexpression in trypanosomes conferred protection from exogenous H_2_O_2_, thus suggesting their role in detoxifying the secondary products of lipid peroxidation reactions (Wilkinson and Kelly, 2003). However, the relevance of nsGPx in the infective stage of *T. cruzi* is unknown.

Finally, cytosolic tryparedoxin peroxidase (cTXNPx), FeSODB1, and GPx-I maintain the redox balance in parasite's cytoplasm. Experiments with recombinant enzyme and FeSOD-overexpressing parasites revealed its importance in virulence and pathogenicity, safeguarding the parasite from host-derived cytotoxic oxidants (Piacenza et al., 2008; Arias et al., 2013; Martínez et al., 2019). The expression levels of cTXNPx as well as of mTXNPx are increased in infective and intracellular stages of the parasite (Zago et al., 2016); these two enzymes work in concert to control the cytosolic and mitochondrial oxidative stress (De Figueiredo Peloso et al., 2011), and are extensively studied for the design of anti-parasite therapies. Lastly, annotation of the parasite genome has revealed that *T. cruzi* lacks catalase, glutathione reductase, thioredoxin reductase, and selenium-dependent glutathione peroxidases (El-Sayed et al., 2005).

### Tryparedoxin Intermediates

An important mediator for the T[SH]_2_-fuelled redox reactions is the dithiol protein and oxidoreductase named tryparedoxin (TXN) (Lopez et al., 2000). In *T. cruzi*, two isoforms TXN-I and TXN-II, have been identified. TXNs are considered members of the thioredoxin family of proteins based on the comparative sequence analysis, presence of common motif WCPPC of the oxidoreductase superfamily in their catalytic center and some other features that are conserved throughout this type of oxidoreductases. However, TXNs display many differences from the host thioredoxins, and are considered to be unique proteins of trypanosomatids and appropriate targets for anti-parasite drug design (Comini et al., 2007). TXNs transfer reducing equivalents from T[SH]_2_ to redox pathway that include cTXNPx, mTXNPx, and nsGPx-I involving thiol/disulfide exchange (reviewed in Arias et al., 2013). In *T. cruzi*, TXN-I is localized in cytosol, and TXN-II is anchored to outer membrane of mitochondria, ER, and glycosomes via a C-terminal hydrophobic tail (Arias et al., 2013). TXN-II is also able to transfer reducing equivalents to low molecular weight disulfides (e.g., GSSG, cystine, dehydroascorbate) and catalyze the reduction of S-nitrosoglutathione and S-nitrosocysteine, and thus, regenerate the reduced form of key metabolites in *T. cruzi* (Arias et al., 2013).

Besides their role in oxidant recycling, new possible functions of TXNs were delineated by interactomics. For example, TXN-I was predicted to interact with other oxidative metabolism components, cysteine and methionine related pathways, as well as protein translation and degradation partners (Piñeyro et al., 2011b). For TXN-II, different interactors were identified under physiological and oxidative stress conditions, and it was suggested to influence the proteins involved in energy metabolism and cytoskeleton and protein translation (Arias et al., 2015; Dias et al., 2018). Further studies will be required to demonstrate the biological significance of TXNs in *T. cruzi* adaptation to different environmental conditions imposed by its life cycle.

## Redox-Dependent Processes

**Figure 2:**
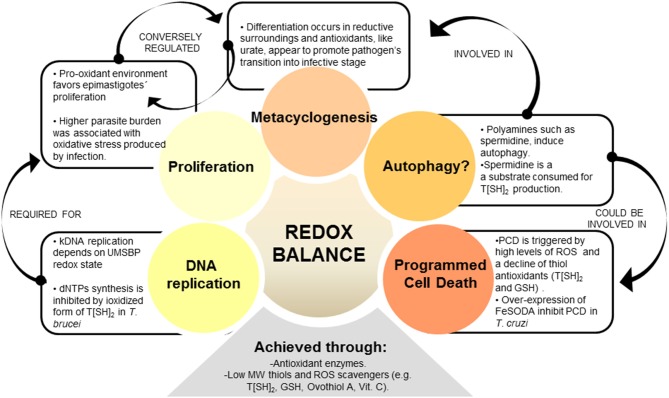
REDOX balance influences different parasite processes. Redox homeostasis is controlled by parasite's antioxidant network, which is formed by enzymatic and non-enzymatic-antioxidant molecules. Among the redox-influenced processes, parasite cell cycle was shown to be regulated by redox balance, i.e., epimastigote's proliferation being induced by oxidative environment. Genomic DNA (gDNA) and kinetoplastid (kDNA) synthesis, as part of cell cycle, are also regulated by redox state in trypanosomatids. Metacyclogenesis is another redox-regulated phenomenon, and some evidence suggest that antioxidants may promote epimastigotes' differentiation to metacyclic infective stage. Besides, over-expression of antioxidant enzyme FeSOD in *T. cruzi* offered enhanced parasite survival through control of programmed cell death (PCD). Autophagy, a self-degradative process for the removal of organelles and proteins, could also be related to redox balance in parasites. Autophagy is triggered by polyamines used as substrate by antioxidant enzymes, and this process is suggested to be associated with metacyclogenesis.

In recent years, several scientists have identified new roles of redox homeostasis. It is suggested that ROS (e.g., H_2_O_2_) at low levels serve as second messenger, and low MW thiols monitor the cellular redox state and play a decisive role in triggering physiological pathways like programmed cell death and DNA replication (Remacle et al., 1995; Gonzalez-Gonzalez et al., 2017; Lorenzen et al., 2017; Phull et al., 2018). Besides, thiol peroxidases appear to be important players in redox signaling processes. In yeast, it is further demonstrated that these enzymes are involved in active peroxide signal distribution via disulfide exchange (Delaunay et al., 2002; Veal et al., 2004; Okazaki et al., 2005; Iwai et al., 2010). Similarly, in mammals, there are several examples showing that peroxiredoxins (PRDXs) function as peroxide receptors and transduce signal to target protein circuits involved in cell growth, differentiation, apoptosis, and carcinogenesis (Neumann et al., 2003; Han et al., 2005; Edgar et al., 2012; Jarvis et al., 2012; Kil et al., 2012; Sharapov and Novoselov, 2019). Yet, it is not fully elucidated how peroxidase-dependent mechanisms would operate in *T. cruzi*. Despite the knowledge gaps, antioxidant network not only protects the parasite from ROS and RNS, but also supports cellular redox-regulated processes (Figure 2).

### Programmed Cell Death (PCD)

Readers are referred to excellent recent reviews describing the signaling events and methods of cell death in higher eukaryotes (Green and Llambi, 2015; Weinlich et al., 2017; D'Arcy, 2019). Briefly, cell death/cell removal occurs by three main modes: apoptosis (type I), autophagy (type II), and necrosis (type III) (Green and Llambi, 2015). In particular, apoptosis describes the death of a cell mediated by an intracellular program that can be triggered by exogenous or endogenous stimuli. Some common features of apoptosis include (a) translocation of phosphatidylserine to outer side of the plasma membrane, (b) annexin and calreticulin exposure on the cell surface, and (c) morphological changes, including condensation of the cell cytoplasm, nuclear DNA fragmentation, dissociation of cell organelles, and disruption of the cell plasma membrane. The boundary between type I and type II cell death is not entirely clear; apoptosis may begin with autophagy (or *vice versa*), and blockage of caspase activity may lead to cell death by type II mode than the type I mode. The switch between type I and type III modes of cell death is determined by a variety of factors, discussed elsewhere (Elmore, 2007; Weinlich et al., 2017). Other types of regulated cell death include necroptosis, pyroptosis, ferroptosis, phagoptosis, and entosis (reviewed in D'Arcy, 2019). Moreover, new insights into these processes show that the border between life and death is so tight that some of these unconventional pathways can in fact promote survival. Remarkably, it was also recently described that cells can recover from the brink of apoptosis or necroptosis through new pathways defined as resuscitation and anastasis (Gudipaty et al., 2018).

Trypanosomes exhibit several features of eukaryotic cell apoptosis, including loss of mitochondrial membrane potential, cytochrome c release, cell shrinkage, and DNA fragmentation, although the exact mechanisms involved in PCD of trypanosomatids and its biological relevance is still under debate (De Souza et al., 2010; Dos Anjos et al., 2016; Menna-Barreto, 2019). The classical caspases and Bcl-2 homologs were not found in trypanosomes, but meta-caspases (T*c*MCA3 and T*c*MCA5) were described in *T. cruzi* (Laverriere et al., 2012). T*c*MCA5-overexpressing *T. cruzi* epimastigotes exhibited apoptosis-like phenotype in presence of fresh human serum (Kosec et al., 2006). Further, *T. cruzi* epimastigotes incubated in fresh human serum exhibited a decline in T[SH]_2_ and GSH levels, decreased mitochondrial aconitase activity, and increased ROS levels (Piacenza et al., 2007). Others have suggested that complement deposition on parasite membranes leads to assembly of membrane attack complex (MAC) that triggers Ca^2+^ influx/overload, disruption of mitochondrial membrane potential, increase in mtROS production, and release of mitochondrial molecules into cytosol, thus signaling apoptosis and parasite death (Irigoín et al., 2009). Further, a recent study showed that cardiomyocytes produce diffusible redox mediators (e.g., H_2_O_2_) that promote PCD in amastigotes and control intracellular proliferation of parasite (Estrada et al., 2018). However, the expression of FeSODA antioxidant is increased in infective trypomastigote and replicative amastigote forms (Atwood et al., 2005), and it is possible that parasite utilizes these antioxidants to evade death in the host cells. Indeed, experimental overexpression of FeSODA arrested PCD in *T. cruzi* (Piacenza et al., 2007), thus providing an experimental proof for the relationship between PCD and antioxidant status in *T. cruzi*. Summarizing, these studies suggest that parasite utilizes antioxidant system to survive in the bloodstream and increase its chances to invade and replicate in the cells of a vertebrate host. Future studies will reveal the mechanistic utilization of antioxidant network by *T. cruzi* to prevent its own death in bloodstream and/or in phagocytes and non-immune cells.

### Degradation Machineries

Eukaryotic cells utilize autophagy to degrade unwanted, aged or damaged cellular material and release the nutrients (e.g., amino acids and nucleotides) for self-preservation. Currently, >40 AuTophaGy-related (ATG) proteins and other proteins that participate in autophagy are known (Xie and Klionsky, 2007; Inoue and Klionsky, 2010; Jin and Klionsky, 2014; Kuma et al., 2017). The autophagy pathway involves several steps including, (1) autophagy induction, (2) cargo selection and package, (3) vesicle nucleation, (4) vesicle expansion and completion, (5) retrieval, (6) fusion of the autophagosome with lysosome vacuole, and (7) vesicle breakdown (Zhao and Zhang, 2018). The readers are referred to excellent reviews for further details of this pathway (Xie and Klionsky, 2007; Chun et al., 2018).

In trypanosomes, though genes involved in cargo packaging are not identified, homologs, or orthologs of >50% of the ATG genes have been predicted by genomic searches (Brennand et al., 2012). Several of these, including TOR1 and TOR2 involved in first step of autophagy (Barquilla and Navarro, 2009) and ATG3, ATG4, ATG5, ATG7, ATG10, and ATG12 involved in vesicle expansion and completion step were characterized in trypanosomatids by a gene knock-out approach (reviewed in Brennand et al., 2012). *T. cruzi* epimastigotes subjected to serum deprivation showed autophagic characteristics such as accumulation of monodansylcadaverine-labeled vesicles and redistribution of TcATG8 (Jimenez et al., 2008). Of the two isoforms of TcATG8 (TcATG8.1 and TcATG8.2), only TcATG8.1 was a *bona fide* ATG8/LC3 homolog that localized in autophagosome-like vesicles in nutrient-starved *T. cruzi* (Alvarez et al., 2008). Vanrell et al. (2017) found that *Tc*ATG8.1 expression and mTOR-dependent autophagy were induced during early stages of metacyclogenesis and showed that spermidine and related polyamines positively regulated autophagy and differentiation of epimastigote to metacyclic trypomastigote form (Vanrell et al., 2017). Spermidine is essential for parasite proliferation (González et al., 2001) and it is also a key metabolite used for T[SH]_2_ synthesis. Others have shown the peroxide concentrations (vs. antioxidant status) direct the parasite differentiation and metacyclogenesis (Nogueira et al., 2015). Together, these findings suggest that oxidants/antioxidants balance and nutrients availability guide the parasite to induce autophagy and metacyclogenesis, the two closely related processes. Specifically, a fine balance of intracellular spermidine and ROS levels likely determine proliferation, autophagy, differentiation to metacyclic trypomastigote form, as well as antioxidant capacity while the parasite gets ready to enter the mammalian host. How parasite senses spermidine and ROS levels for such a fine-tuned cell cycle control is still unknown.

Eukaryotic cells also utilize several additional approaches, including ER-associated degradation (ERAD), lysosomal proteases, and 20S proteasome complex to remove the oxidized/degraded proteins (reviewed in Korovila et al., 2017; Hwang and Qi, 2018). Trypanosomes lack an elaborated ERAD network to recycle their proteins (Harbut et al., 2012). Ubiquitin proteasome pathway (UPP)—an alternative system for the turnover of damaged proteins—is expected to be present based on the finding of 269 putative components of the UPP pathway in *T. cruzi* proteome (Gupta et al., 2018). Some of the UPP components are unique to *T. cruzi*, and are proposed to be potential drug targets (Gupta et al., 2018). Specifically, proteasome activity is detected in all life cycle stages of *T. cruzi*, and its inhibition increased the carbonylated protein content, resulting from formation of covalent, non-reversible lipid aldehyde adducts on side chain of cysteine, histidine, and lysine residues. Others have shown that treatment with specific proteasome inhibitors blocked *T. cruzi* growth, metacyclogenesis (Cardoso et al., 2008), and intracellular amastigote-to-trypomastigote differentiation (González et al., 1996). Indeed, proteasome-dependent proteolysis of carbonylated proteins that were extensively present in late log phase epimastigote cultures was suggested to signal metacyclogenesis (Cardoso et al., 2011). Thus, it appears that ROS induction of protein damage and protein degradation machinery might play an important role in parasite development, though further in-depth studies are needed to delineate the mechanistic role of ROS in regulating protein degradation machineries in trypanosomes.

### Nucleotides Synthesis and DNA Replication

Cellular DNA synthesis and proliferation, as well as DNA repair mechanisms depend on the production of a balanced supply of deoxyribonucleotides (dNTPs). Ribonucleotide reductases (RNRs) catalyze the *de novo* synthesis of dNTPs from the corresponding ribonucleotides. Though RNRs are highly diverse, their catalytic activity requires peptides harboring a free radical, redox-active thiols, and proteins of the thioredoxin family, and, therefore they are directly influenced by cell redox environment. Indeed, a tight relationship between ROS level and DNA replication is described in higher eukaryotes (Shackelford et al., 2000; Burhans and Heintz, 2009). Macrophage production of NO and NO donors inhibited DNA synthesis, likely through NO-mediated inactivation of critical thiols on R1 subunit and destruction of tyrosyl radical on the R2 subunit of RNRs (Holmgren and Sengupta, 2010). Others showed that increased levels of ROS dissociate replication accelerator-component from the replisome complex and slow down DNA synthesis as a safeguard for genome stability (Somyajit et al., 2017). Importantly, it was shown that peroxiredoxin-2 (PRDX2), an antioxidant enzyme, forms a replisome-associated ROS sensor to regulate the replication machinery (Somyajit et al., 2017), pointing out the relevance of the redox signaling/antioxidant status in dNTP synthesis and DNA replication.

The finding of an inhibitory effect of oxidized forms of T[SH]_2_ and tryparedoxin on dNTP synthesis in *T. brucei* (Dormeyer et al., 2001) offered the initial evidence for a relationship between *de novo* nucleotide synthesis and redox status in trypanosomes. Authors noted that tryparedoxins transfer the reducing energy from T[SH]_2_ to RNR enzyme (Dormeyer et al., 2001). In *T. cruzi*, we observed a direct correlation between the intracellular T[SH]_2_ content and replication of epimastigotes (Mesías et al., 2018). These observations suggest that T[SH]_2−red_/T[SH]_2−ox_ ratio, at least partially, regulates the DNA synthesis during parasite proliferation. How the oxidant/antioxidant balance could modulate genomic DNA replication inside parasite nucleus remains to be understood in future studies.

Replication of parasite's kinetoplast DNA (kDNA) is also dependent on redox status. Kinetoplast is a unique DNA structure present in a single large mitochondrion located close to the nucleus of trypanosomatids. Each kinetoplast consists of 10–20 copies of maxicircle DNA (20,000–40,000 bp) and 10,000 or more copies of topologically interlocked minicircle DNA (<1,000 bp). Like mtDNA, maxicircles encode rRNAs and components of the respiratory complexes. The heterogeneous sequences of minicircle DNA encodes guide RNAs that function in mRNA editing process (reviewed in Cavalcanti and De Souza, 2018). The replication of minicircle DNA is a complex mechanism that begins with the attachment of the universal minicircle sequence (UMS), located at the origin of replication of minicircle DNA, with the UMS binding protein (UMSBP), firstly identified in *Crithidia fasciculata* (a related, non-pathogenic trypanosome) (Tzfati et al., 1992) and more recently in *T. cruzi* (Coelho et al., 2003). Recent studies showed that UMSBP affinity for target kDNA and oligomerization was sensitive to redox potential in *C. fasciculata* and *Leishmania donovani* (Onn et al., 2004; Singh et al., 2016), and the reduction of UMSBP activates its binding to the minicircle DNA origin site, whereas UMSBP oxidation impaired this activity. Molecules of the antioxidant network, TXN-II and TXNPx, were found to be involved in governing the oxidized/reduced state of the UMSBP in *C. fasciculata* (Sela et al., 2008). Further, deletion of UMSBP in *Leishmania* resulted in decreased ATP production associated with reduced complex III activity (Singh et al., 2016), an event that also results in increased electron leakage and superoxide generation in mitochondria (Wong et al., 2017).

Thus, parasite's redox environment, modulated by its antioxidant machinery, regulates dNTP synthesis, kDNA replication, and oxidative phosphorylation that are necessary for trypanosomes proliferation.

## Possible Fine-Tuning Mechanisms of *T. cruzi* Antioxidant System

As discussed above, *T. cruzi* is constantly exposed to ROS and RNS throughout its life cycle and needs to effectively coordinate the antioxidant and repair systems to overcome the toxic effects of oxidative stress. These responses ought to be precise enough to control the effects of the exogenous insult without altering the cellular environment in the parasite, as this can affect other cell signaling pathways. Even more, parasite has to assemble specific responses according to the type of oxidative insult. This section is focused on what we know, and mostly, what we do not know about how trypanosomes achieve fine-tuning of the antioxidant system.

**Figure 3:**
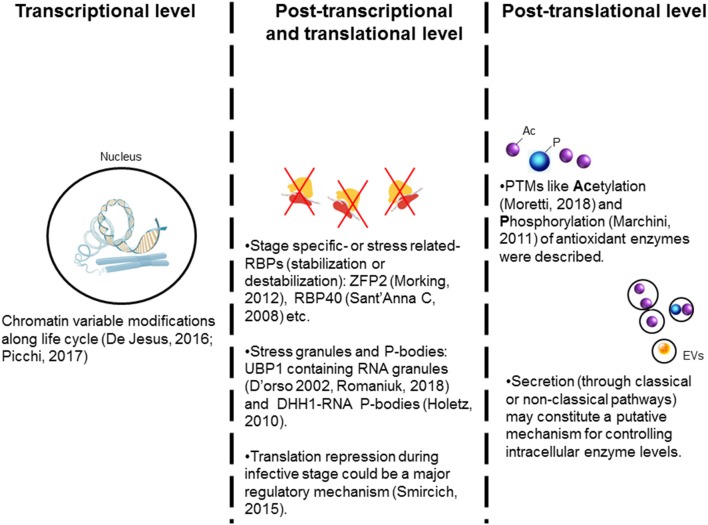
Possible mechanisms controlling the antioxidant enzymes' expression in trypanosomes. The expression of many of the antioxidant enzymes is increased during metacyclogenesis and in infective stage suggesting that stage-specific regulation mechanism(s) are involved. (1) Stage-specific post-translational modifications (PTMs) of histones are suggested to offer transcriptional regulation of antioxidants' expression in different parasite life cycle stages. (2) RNA binding proteins (RBP) are suggested to positively or negatively target the mRNA's stability and offer post-transcriptional regulation. The RNA-granules containing ribosomal machinery are suggested to arrest the translation of mRNAs for antioxidants. Overall, translational repression appears to be a general response during stress and in the infective stages. (3) Post-translational modifications, especially acetylation and phosphorylation, of antioxidants may provide a tight regulation of the enzymatic activity. Further, we propose that secretion of the antioxidants with extracellular vesicles (EV) acts as a mechanism to control intracellular concentration of antioxidants in the parasite.

The unbiased transcriptomic and proteomic approaches have shown that the expression and activity of the enzymes of the antioxidant network are up-regulated in the infective and intracellular stages of *T. cruzi* (Atwood et al., 2005; Parodi-Talice et al., 2007; Zago et al., 2016). At least partly, one regulation layer should reside on stage-specific mechanisms. Ribosome profiling studies have indicated that *T. cruzi*, during the infective metacyclic stage, exerts a translational control through down-regulation of ribosomal components and up-regulation of virulence factors (e.g., trans-sialidases) (Smircich et al., 2015). Recent advances in chromatin proteomics have revealed a substantial association between histone post-translational modifications (PTMs) and stage-specific transition in *T. cruzi* (De Jesus et al., 2016; Picchi et al., 2017) (Figure 3). What is more interesting, some of the PTMs of histones were not only novel and unusual, such as alternative lysine acetylation, serine/threonine acetylation, and N-terminal methylation, but also some of these PTMs were unique to *T. cruzi* and other trypanosomes (De Jesus et al., 2016; Picchi et al., 2017). Thus, it is likely that as *T. cruzi* undergoes stage-specific transitions in multiple hosts, it employs histone's modifications to organize chromatin along with the traditional post-transcriptional and translational mechanisms to drive the expression and activity of its antioxidant network. This hypothesis remains to be experimentally proven.

In trypanosomatids, gene expression is not regulated at the transcription initiation level (reviewed in Teixeira and DaRocha, 2003; Martínez-Calvillo et al., 2010; De Gaudenzi et al., 2011). Instead, RNA-binding proteins (RBPs) that associate with mRNAs and other regulatory proteins to form ribonucleoprotein complexes (mRNPs) are suggested to exert post-transcriptional regulation in trypanosomes. There are a wide variety of RBPs, displaying different RNA binding domains, e.g., RNA-recognition motif (RRM), pumilio and Fem-3-binding factor (PUF), as well as zinc fingers (CCCH). Several RBPs were described in *T. cruzi* (Alves and Goldenberg, 2016; Romaniuk et al., 2016), and some were suggested to be involved in stabilization (or destabilization) of mRNA in response to extracellular stimuli (Fernández-Moya et al., 2012; Alves and Goldenberg, 2016). Specifically, RBSR1 and the nuclear SR protein TRRM1 were identified as nutritional stress-related RBPs in *T. cruzi* (Wippel et al., 2018). *T. cruzi* may also employ RBPs to form active or repressive mRNP complexes for post-transcriptional regulation of antioxidant machinery in response to other stress sources.

Other regulation layers may involve the microenvironment within the parasite and the exogenous stimuli present in the host environment. For example, RNA stress granules (SGs) were proposed to regulate gene expression in response to local stress (Protter and Parker, 2016; Khong et al., 2017). SGs are ribonucleoprotein assemblies that incorporate different components depending on the stimuli present (e.g., heat shock, starvation, oxidative stress, etc.) to regulate the post-transcriptional fate of mRNAs (Chen and Liu, 2017; Harvey et al., 2017). These cytoplasmic foci act as traps for dispensable mRNAs, and thereby, prevent new translation initiation until the stress factor(s) have been removed (Rodrigues et al., 2010). The occurrence of mammalian-like SGs and protein synthesis arrest by eIF2α phosphorylation in response to stress was described in *T. cruzi* (Tonelli et al., 2011). Further, uridine-binding protein 1 and 2 (UBP1 and UBP2) that are present in the nucleus in small amounts under normal conditions, may accumulate in the cytoplasm under oxidative stress to form RNA SGs and affect mRNA translation (Cassola et al., 2007). Recent studies have demonstrated UBP1-dependent translational repression as a mechanism for stage-specific regulation of gene expression in *T. cruzi* (Romaniuk et al., 2018) (Figure 3). DHH1, a DEAD box RNA helicase, is a common component of both SGs and processing bodies (P-bodies, constitutive RNA-granules); and it was described in *T. cruzi* (Holetz et al., 2007). Interestingly, DHH1-containing granules in *T. cruzi* were found to comprise a putative TryS-coding transcript (Holetz et al., 2010), thus indicating that SGs (and P-bodies) may also regulate the antioxidant response in *T. cruzi*.

In higher eukaryotes, phosphorylation, acetylation, and ubiquitination are usually ROS-sensitive PTMs (Liu et al., 2013; Tseng et al., 2014). In *T. cruzi* and other trypanosomatids, PTM-dependent regulation of signaling mechanisms is not studied in detail. Schenkman and co-workers have recently developed the acetylome of *T. cruzi*, and showed that some cytosolic enzymes including antioxidant effectors were continuously acetylated and deacetylated; presumably as a control system for their enzymatic activity or protein-protein interaction (Moretti et al., 2018). Further, a large-scale phosphoproteome analysis study showed that antioxidant enzymes, TryR, TryS, and thiol-dependent reductase 1, are major targets of phosphorylation during the metacyclogenesis transition process (Marchini et al., 2011; Amorim et al., 2017). Thus, PTMs could have a key role in achieving a rapid regulation of antioxidants enzymatic activity as well as in stage-specific modulation of antioxidants expression. Additionally, some antioxidant enzymes are actively secreted by the infective stages of *T. cruzi* (Bayer-santos et al., 2013; Gadelha et al., 2013), and parasite may utilize this secretory pathway to regulate the intracellular protein contents.

To sum up, parasite must elicit a rapid and precise response toward oxidative stress and maintain redox homeostasis. It appears that diverse mechanisms (i.e., chromatin modification, RBPs, and PTMs) are involved in stage-specific regulation of antioxidant gene expression during metacyclogenesis and in infective/replicative forms of *T. cruzi* (Atwood et al., 2005; Parodi-Talice et al., 2007; Zago et al., 2016). In addition, control strategies (RBPs, stress granules conformation, PTMs) may also be employed to fine-tune the adaptation to microenvironment. Future research will provide precise information regarding the specific regulation processes acting over antioxidant components in each situation.

## Conclusions and Remarks

In this review, we have discussed the different oxidative challenges that parasite has to face throughout its life stages, the mechanism(s) employed for controlling this condition, and the cellular processes influenced by the parasite's intracellular redox environment. Further analysis of the interacting partners of the antioxidant components could give us a deeper knowledge of the pathways depending on redox balance. If we consider the available interactomes in trypanosomes (Piñeyro et al., 2011b; Arias et al., 2015; Peloso et al., 2016), it is clear that antioxidant system is linked to many cellular pathways. It is our hope that future investigations will focus on understanding the crosstalk between antioxidants and other cellular processes and enhance our understanding of the influence of redox balance on *T. cruzi* biology.

Different drugs are assayed for the treatment of ChD and sometimes their trypanocidal activity relies on their ability to produce a redox imbalance over the parasite cell. Indeed, nifurtimox and benznidazole, current drugs administered to treat acute ChD, generate a non-specific oxidative effect over pathogen macromolecules. Conversely, several naturally produced antioxidant compounds (e.g., vitamins B12, C, K3, and catechin) were also shown to have a degree of anti-parasitic effect (Paveto et al., 2004; Ciccarelli et al., 2012; Desoti et al., 2015). In some cases, the toxic effects of antioxidants were explained as a consequence of ROS increase, produced by the redox cycling process. In other words, antioxidant administration, like pro-oxidants, may lead to a compromising cell redox imbalance controlling parasite spread.

There is no doubt about the relevance of the antioxidant network for rational drug design against trypanosomatids (Piacenza et al., 2009b; Flohé, 2012; Talevi et al., 2018). The deeper knowledge of the mechanisms involved in the regulation of antioxidant responses in the different life cycle stages of the parasite will provide better opportunities for designing new, multi-component, parasite control strategies.

## Author Contributions

AM conceptualized the study. AM, MZ, and NG searched for literature, wrote the manuscript, reviewed and edited the manuscript.

### Conflict of Interest

The authors declare that the research was conducted in the absence of any commercial or financial relationships that could be construed as a potential conflict of interest.

## Footnotes

^1^World Health Organization (WHO) | Epidemiology, http://www.who.int/chagas/epidemiology/en/

^2^Drugs for Neglected Diseases initiative (DNDi); https://www.dndi.org/achievements/paediatric-benznidazole/
